# Booklet for knowledge and prevention of HIV mother-to-child transmission: a pilot study of a randomized clinical trial*

**DOI:** 10.1590/1980-220X-REEUSP-2021-0560en

**Published:** 2022-11-25

**Authors:** Ana Carolina Maria Araújo Chagas Costa Lima, Sadrine Maria Eufrasino de Pinho, Sabrina Alapenha Ferro Chaves Costa Lima, Anne Fayma Lopes Chaves, Camila Moreira Teixeira Vasconcelos, Mônica Oliveira Batista Oriá

**Affiliations:** 1 Universidade Federal do Ceará, Faculdade de Farmácia, Odontologia e Enfermagem, Departamento de Enfermagem, Fortaleza, CE, Brazil.; 2 Universidade Estadual do Ceará, Centro de Ciências da Saúde, Programa de Residência em Enfermagem Obstétrica, Fortaleza, CE, Brazil.; 3 Escola de Saúde Pública do Ceará, Residência Integrada em Saúde, Saúde da Família e Comunidade, Fortaleza, CE, Brazil.; 4 Universidade da Integração Internacional da Lusofonia Afro-Brasileira, Instituto de Ciências da Saúde, Departamento de Enfermagem, Redenção, CE, Brazil.

**Keywords:** Infectious Disease Transmission, Vertical, HIV, Health Education, Teaching Materials, Nursing, Clinical Trial, Transmisión Vertical de Enfermedad Infecciosa, VIH, Educación en Salud, Materiales de Enseñanza, Enfermería, Ensayo Clínico, Transmissão Vertical de Doença Infecciosa, HIV, Educação em Saúde, Materiais de Ensino, Enfermagem, Ensaio Clínico

## Abstract

**Objective:**

To test the effectiveness of the booklet, compared to the usual service care, in the increase of the knowledge of pregnant/puerperal women living with HIV, for the prevention of HIV-VT.

**Method:**

Pilot study of a randomized controlled clinical trial, initially with 104 pregnant women living with HIV, with a final sample of 45 women. It was held in three public maternity hospitals in Fortaleza-CE, from January/2017 to May/2018. The control group received regular care from the service and the intervention group had access to the booklet as an additive. The research was carried out in three phases: baseline; evaluation 2, in prenatal care; and evaluation 3, in the postpartum period.

**Results:**

There was no intergroup difference in the women’s mean knowledge score (short-term p = 0.473; long-term p = 0.151). However, in the intragroup analysis, the booklet proved to be effective in improving the pregnant women’s knowledge in the intervention group, in the short term (p = 0.002) and long term (p = 0.033).

**Conclusion:**

There was an improvement in knowledge within the intervention group over time, but there was no difference in women’s knowledge in the intergroup analysis. Thus, based on this pilot, a broader study on the use of booklet is required to prove its effectiveness (ReBEC: UTN: U1111-1191-9954).

## RESUMO

**Objetivo:**

Testar efetividade de cartilha, em comparação ao atendimento habitual do serviço, no aumento do conhecimento de gestantes/puérperas que vivem com HIV, para prevenção da TV-HIV.

**Método:**

Piloto de ensaio clínico randomizado controlado, inicialmente com 104 gestantes que vivem com HIV, com amostra final de 45 mulheres. Realizado em três maternidades públicas de Fortaleza-CE, de janeiro/2017 a maio/2018. O grupo controle recebeu atendimento habitual do serviço e o grupo intervenção teve como aditivo acesso à cartilha. A pesquisa foi realizada em três fases: linha de base; avaliação 2, no pré-natal; e avaliação 3, no pós-parto.

**Resultados:**

Não houve diferença intergrupos na média da pontuação do conhecimento das mulheres (curto prazo p = 0,473; longo prazo p = 0,151). Porém, na análise intragrupo, a cartilha se mostrou efetiva para melhorar o conhecimento das gestantes do grupo intervenção, em curto prazo (p = 0,002) e longo prazo (p = 0,033).

**Conclusão:**

Houve melhora do conhecimento dentro do grupo intervenção, ao longo do tempo, porém não foi evidenciada diferença quanto ao conhecimento das mulheres na análise intergrupos. Assim, a cartilha carece da realização de estudo mais amplo, a partir deste piloto, para comprovação de sua efetividade (ReBEC: UTN: U1111-1191-9954).

## RESUMEN

**Objetivo:**

Testar la eficacia del cuardenillo, en comparación al servicio habitual, en el incremento del conocimiento de las mujeres embarazadas/puérperas que viven con el VIH, para la prevención del TV-VIH.

**Método:**

Piloto de un ensayo clínico controlado aleatorizado, inicialmente con 104 mujeres embarazadas que viven con el VIH, con una muestra final de 45 mujeres. Realizado en tres maternidades públicas de Fortaleza-CE, de enero/2017 a mayo/2018. El grupo control recibió atención regular del servicio y el grupo intervención tuvo acceso al cuadernillo como complemento. La investigación se llevó a cabo en tres etapas: línea de base; evaluación 2, en atención prenatal; y evaluación 3, en el puerperio.

**Resultados:**

No hubo diferencia entre grupos en la puntuación media de conocimiento de las mujeres (a corto plazo p = 0,473; a largo plazo p = 0,151). Sin embargo, en el análisis intragrupo, el cuardenillo se mostró eficaz en la mejora del conocimiento de las gestantes del grupo intervención, a corto plazo (p = 0,002) y a largo plazo (p = 0,033).

**Conclusión:**

Hubo una mejora en el conocimiento dentro del grupo de intervención con el tiempo, pero no hubo diferencia en el conocimiento de las mujeres en el análisis intergrupal. Por lo tanto, el cuadernillo necesita realizar un estudio más amplio, basado en este piloto, para probar su efectividad (ReBEC: UTN: U1111-1191-9954).

## INTRODUCTION

In the last ten years, the epidemiological scenario of Human Immunodeficiency Virus (HIV) infection has shown a high number of cases of women of childbearing age, with a 38.1% increase in the detection rate for pregnant women with HIV^(1)^. The Vertical Transmission of HIV (HIV-VT) takes place through the transmission of the virus from the mother to the baby, during pregnancy, labor, delivery itself, or breastfeeding. In planned pregnancies, with interventions carried out properly from prenatal care to the puerperium and the exposed newborn, the risk of vertical transmission of HIV is reduced to less than 2%^(2,3)^.

Therefore, a set of measures is required to minimize the risks^(2)^. However, despite the high effectiveness of prophylaxis to reduce the chances of vertical transmission, studies show important flaws in the care cascade of infected pregnant women that hinder the reduction of mother-to-child HIV transmission rates: late diagnosis of infection during pregnancy; failure to provide counseling and guidance to all women during prenatal care; failure to use Antiretroviral Therapy (ART) properly; the lack of organization of health services, as well as poor knowledge on the part of pregnant women in relation to preventive measures^(4,5,6,7)^.

It appears that pregnant women have gaps in understanding about HIV, the forms of transmission, tests, and how to use ART correctly, which shows the need to improve the process of health education carried out by health professionals, aiming at reducing the consequences of the disease^(8)^. Thus, to promote the learning process, professionals have implemented the use of educational technologies, which can favor behavioral changes, making the client confident to carry out a certain health-promoting behavior^(7,9)^.

Upon understanding the importance of these aspects, an educational booklet entitled “How to prevent mother-to-child HIV transmission? Be up on it!” was developed and validated with specialists on the subject and with pregnant and postpartum women living with HIV, which aims to promote greater autonomy for women living with HIV who are in the pregnancy-puerperal period regarding care for the prevention of HIV-VT^(9)^.

Through the use of this booklet, in health education actions, it is possible to establish a co-participatory and dialogic relationship between nurses and pregnant women living with HIV, providing better knowledge and greater empowerment to carry out the recommended care for the prevention of vertical transmission. In addition, the use of this booklet will enable the use of technology that can direct, standardize, systematize, and streamline the educational actions carried out by health professionals, especially nurses, in the approach to HIV-VT prevention and health promotion of the mother-child binomial.

Therefore, we aimed to test the educational booklet effectiveness, compared to the usual service, in the increase of the knowledge of pregnant/puerperal women living with HIV, for the prevention of HIV-VT.

## METHOD

### Design of Study

This is a Pilot Study of a Randomized Controlled Clinical Trial (RCT). To report the study, we followed the *Consolidated of Reporting Trials* (CONSORT) *for Randomized Trials of Nonpharmacologic Treatments* ^(10)^. Pilot studies guide decisions on how to design recruitment approaches, measurements and interventions, being beneficial in studies addressing new intervention^(11)^. Considering that this clinical trial addresses the evaluation of unprecedented educational technology in the subject addressed, the performance of a pilot study was chosen before carrying out a larger-scale RCT.

The PICO strategy – acronym for **P**atient, **I**ntervention, **C**omparison and **O**utcomes – was followed. This strategy is widely used for the elaboration of research questions, in which the first element (P) consists of pregnant and postpartum women living with HIV; the second (I) is represented by the application of the educational booklet; the third (C) used, in the comparative group, the service usual outpatient care; and the fourth (O) refers to the increased knowledge about HIV-VT prevention.

### Population and Local

The study was carried out between January 2017 and May 2018, in three public maternity hospitals located in the city of Fortaleza-CE, Brazil. The sample consisted of pregnant women living with HIV who were undergoing prenatal care at the chosen institutions, during the data collection period, who met the pre-established criteria, agreed to participate in the study, with the proper signature of the free and informed consent form, and completed the follow-up.

### Selection Criteria

The following inclusion criteria were used: being pregnant with proved HIV, regardless of gestational age, chronological age and time of HIV diagnosis; being on prenatal care in the chosen institutions during the collection period; having telephone contact. Pregnant women with compromised physical or mental health were excluded, as well as illiterate women, as this could be a confounding variable. The following were considered discontinuity criteria: withdrawal from participating in the research after the start of data collection; delivery before the second evaluation; death or abortion during the study period; no return to service after baseline or phone number change; not answering phone calls or having the phone turned off, after three attempts, on consecutive days and at different times.

A total of 104 mothers living with HIV were elected; however, there was a 56.7% loss, despite the researchers having adhered to the data collection protocol, seeking to minimize them. Thus, the final sample consisted of 45 women, with 24 in the Intervention Group (IG) and 21 in the Control Group (CG).

To assess whether the losses were random, sociodemographic data (marital status, race, religion, current occupation, age, education, and income) collected at the beginning of the study were analyzed and pregnant women who were followed up to the end of the study were compared with those lost to follow-up. Thus, the groups were evaluated for homogeneity, using the z test of proportions, and it was observed that there was no statistically significant difference, since the null hypothesis was confirmed, with a significance value of 5% being considered. Thus, it was found that the losses were random.

The sample was randomly allocated to the two groups. The pregnant women who participated in the CG received usual outpatient care from the service (individualized verbal guidance during prenatal consultations with health professionals: doctors, nurses, and social workers). The Intervention Group (IG) had access to the previously constructed and validated booklet in the three health institutions of the study^(9)^.

### Data Collection

**Figure 1.**
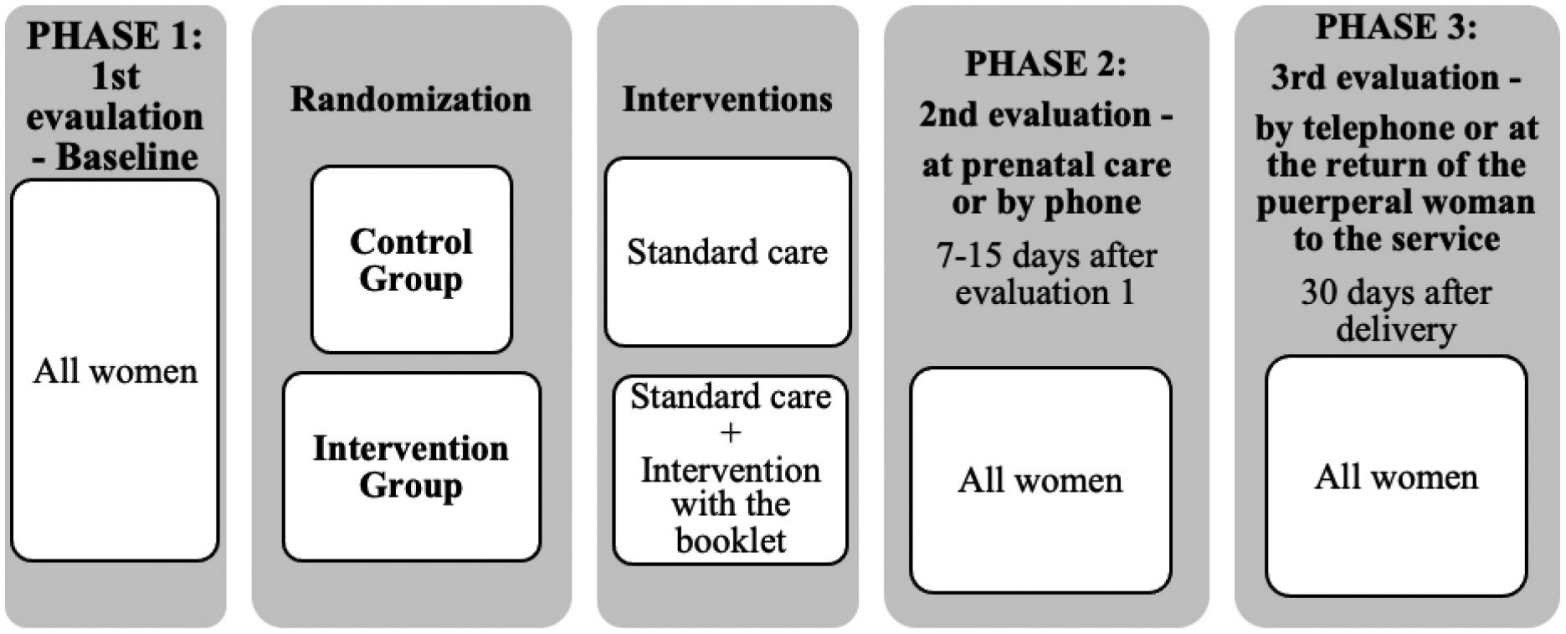
RCT methodological flowchart – Fortaleza, CE, Brazil, 2018.

The research was carried out in three phases, as shown in Figure 1.

In the first evaluation, baseline, the collection instrument was applied to all pregnant women, regardless of the group, at the prenatal consultation site. Then, the groups were randomized, which was done in blocks (block randomization). A table with a sequence of randomized numbers was previously generated for both the control group and the intervention group, in blocks of 10 women for each group, using a computer software, through the website http://www.randomizer.org. Thus, after collecting data from the baseline women, the person in charge for randomization drew a number from an opaque envelope and this number corresponded to one of the groups. Then, the color of the group to which the patient belonged was marked with a pen on the kit with the collection instruments and on the study follow-up worksheet.

On the same day, the women who belonged to the intervention group went to a reserved place, where a researcher was waiting for them with the booklet for the educational intervention. The meeting took place once, considering that afterwards the pregnant women would take the booklet with them and could consult it whenever necessary. The intervention took place individually and was applied by the principal investigator or by a properly trained nurse member of the research team, lasting around 15-20 minutes.

To implement the educational approach, the booklet “How to prevent mother-to-child HIV transmission? Be up on it!”, which has as its content basic information about HIV and the main care recommended for the prevention of HIV-VT, during pregnancy (use of medication; attendance at prenatal consultations; periodic examinations; importance of maintaining a healthy lifestyle); delivery (possible types of delivery and the use of intrapartum medication); and puerperium (use of medication by the child; non-recommendation of breastfeeding; availability of free milk formula up to six months of age; and the importance of monitoring the mother and child in a specialized service)^(9)^.

Initially, the booklet and its purpose were presented, raising awareness among pregnant women about the need to prevent HIV-VT. The possibility of interruption was agreed in case of doubts or comments. Then, the booklet was read together with the pregnant woman. This same type of educational approach was implemented in a clinical trial with printed educational material, with positive results^(12)^.

The second evaluation took place through telephone call, between seven and 15 days after the first evaluation, which sometimes coincided with the day of the consultation subsequent to the first evaluation. The third evaluation took place 30 days after delivery. At this stage, data collection took place through phone calls or during the birth review consultation. The telephone collection took an average of 15 minutes, and the woman was asked to look for a reserved place for the interview during the call. To have better control as to the dates for follow-up of women in both the control and intervention groups, a worksheet was created to monitor the stages of the study.

There was blinding only of the researchers responsible for the follow-up evaluations, for typing the database and the statistics. However, neither the women participating in the study nor the researchers responsible for the application of the intervention and randomization could be blinded, as it was an educational intervention.

To standardize the information, as well as minimize possible biases, training was carried out with the team responsible for data collection and application of the intervention. The stages of the study were implemented by different people, being kept “blind” as to the other stages.

An instrument was developed to assess the knowledge of women in relation to care for the prevention of HIV-VT, which underwent a process of validation of appearance and content with expert judges in the thematic area before being applied.

The instrument contains the first part for sociodemographic data (age, origin, education, occupation, income, number of people in the house, marital status, race, religion) and reproductive history of pregnant women; and the second part regarding knowledge about HIV-VT prevention, with four open questions: 1. Have you heard about care to prevent mother-to-child transmission of HIV? (varies from 0–1 point, with 1 point for “yes” and 0 for “no”); 2. Do you know the precautions to prevent transmission of HIV to your child during pregnancy? (0–7 points); 3. Do you know the precautions to prevent transmission of HIV to your child at birth? (0–3 points); 4. Do you know the precautions to prevent the transmission of HIV to your child in the postpartum period? (0–5 points).

Each of the four questions was asked openly and, for each care mentioned as known, what the patient reported was marked, with a point being assigned for each care correctly informed. For example, in the question “Do you know the precautions to be taken to prevent the transmission of HIV to your child during pregnancy?”, if she answered “use of medication against HIV”, a point was assigned; if she mentioned another correct care, a point was awarded; if she did not know how to answer, she did not score on that item. Thus, there was a list of care that could be mentioned and there was open space for new responses. In the same way, the subsequent questions followed. At the end, the score was added, which could range from zero to 16 points, investigating the pregnant women’s prior knowledge, to compare the knowledge before and after the intervention with the booklet.

Thus, knowledge regarding HIV-VT was evaluated, regarding care to prevent HIV-VT in pregnancy, childbirth, and after the child’s birth, and whether the increase in knowledge was statistically significant in the intra and intergroups was checked. Regarding the knowledge score, as it is an instrument built and validated in the study itself and never applied to this public before, it was not possible to measure satisfactory levels of knowledge. The results presented can be used as a basis for future research.

### Data Analysis and Treatment

The data obtained were compiled in the statistical software *Statistical Package for the Social Sciences* (SPSS), version 24.0. In the exploratory phase, measures of central tendency (mean) and dispersion (standard deviation) and calculations of simple and relative frequencies were considered. With regard to the inferential phase, bivariate analyses were initially developed for homogeneity, intergroup comparison (intervention × control) and intragroup comparison (before and after). In the bivariate analysis, the following tests were adopted, according to the type of variable and normality: likelihood ratio; Student’s t test for independent samples; Student’s t test for paired data; chi-square.

### Ethical Aspects

The study was approved by the Research Ethics Committees of the institutions where the research was carried out, according to Opinion 1.684.549 (approved in 2016) and 1.930.501 (approved in 2017), being registered on the platform Brazilian Registry of Clinical Trials (ReBEC) (UTN: U1111-1191-9954). All ethical aspects related to research with human beings were respected, in accordance with Resolution No. 466/2012 of the National Health Council. Pregnant women were invited to participate in the study. After reading, together with the researcher, and agreement, they signed the Free and Informed Consent Form (FICF).

## RESULTS

**Figure 2.**
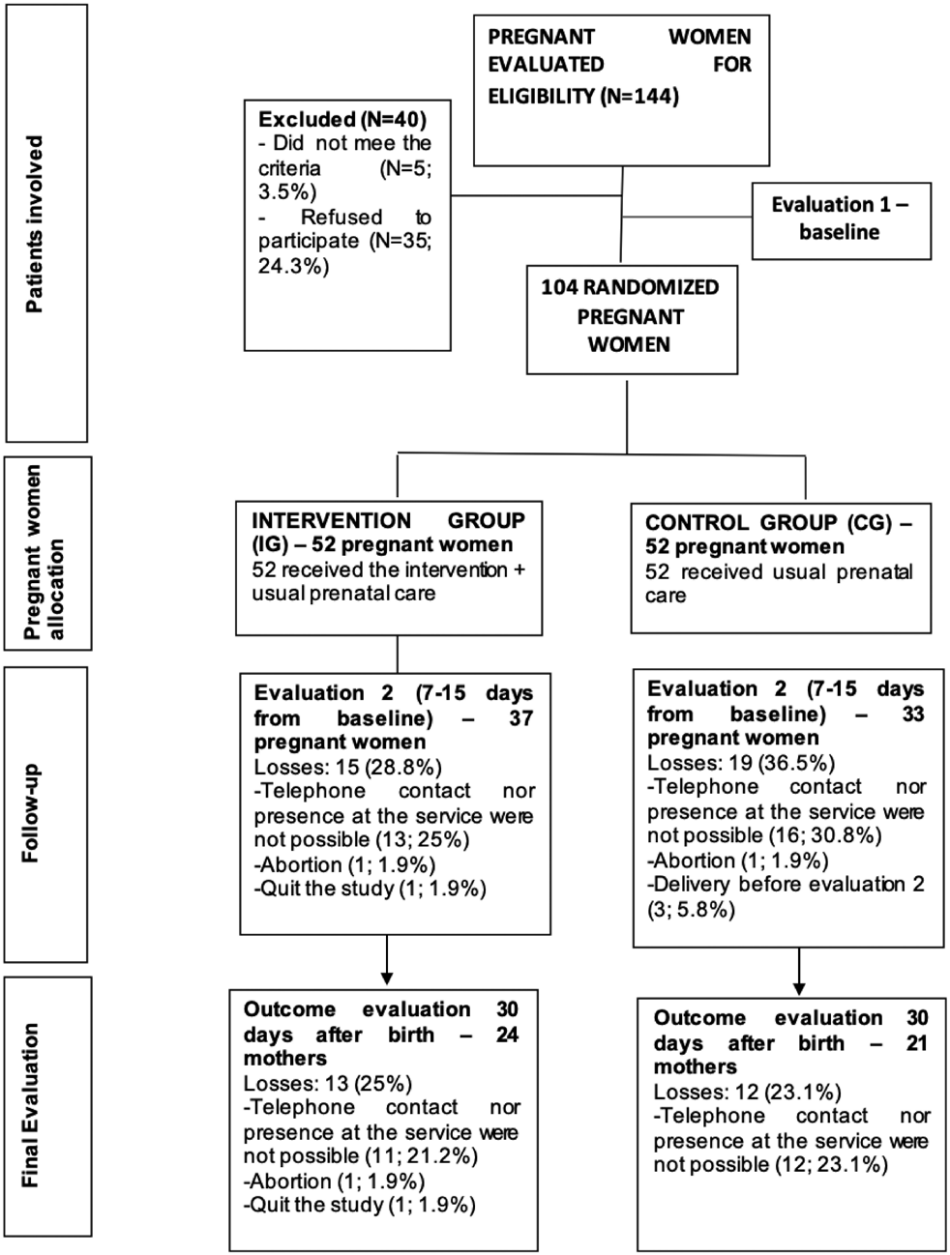
Diagram representing the flow of participants in each phase of the study, as stated in CONSORT – Fortaleza, CE, Brazil, 2018.

In phase 1 of the study, 144 pregnant women were initially evaluated on the day of the prenatal consultation for eligibility, with 104 mothers living with HIV being considered eligible. Figure 2 presents the follow-up of the participants in each phase of the study.

In Table 1, the groups’ homogeneity was verified, based on the sociodemographic data, to ensure that the differences between them did not exceed what could be expected by chance and that the variables did not interfere with the study outcomes.

**Table 1.** Sociodemographic characterization of pregnant women living with HIV – Fortaleza, CE, Brazil, 2018. (n = 104)

		Total (n = 104)				Intervention (n = 52)				Control (n = 52)				p-value
		N	%	Mean	SD*	N	%	Mean	SD*	N	%	Mean	SD*
**Age (years)**				26.91	6.09			27.31	5.98			26.52	6.24	0.512^†^
**Origin**	Fortaleza	58	55.77			30	57.69			28	53.84			0.783^‡^
	Other municipalities in Ceará	41	39.42			19	36.54			22	42.31			
	Other Brazilian states/other countries	5	4.81			3	5.77			2	3.85			
**Education (years)**				9.14	2.47			9.19	2.38			9.10	2.58	0.844^†^
**Occupation**	Housewife	58	55.8			29	55.8			29	55.8			0.774^§^
	Unemployed	10	9.6			4	7.4			6	11.5			
	Other	36	34.6			19	36.54			17	32.7			
**Marital status**	Single/Widow	31	29.81			13	25.00			18	34.62			0.284^§^
	Married/Common law marriage	73	70.19			39	75.00			34	65.38			
**Race**	White	8	7.69			3	5.77			5	9.62			**0.030** ^‡^
	Black	9	8.65			8	15.38			1	1.92			
	Brown	87	83.66			41	78.85			46	88.46			
**Religion**	Catholic	52	50.00			22	42.31			30	57.69			0.208^§^
	Evangelical	37	35.58			20	38.46			17	32.69			
	Other	15	14.42			10	19.23			5	9.62			

*SD = Standard Deviation; ^†^ = Student’s t test for independent samples; ^‡^ = Likelihood ratio; ^§ =^ Chi-square.

Table 1 shows that the intervention and control groups are homogeneous in terms of sociodemographic characteristics of pregnant women, except for race (p = 0.030).

Table 1 shows that the sample profile consisted of young women (M = 26.9 years; SD = ± 6.09), ranging from 15 to 43 years old, with low level of education (M = 9.14 years of study; SD = ± 2.47) and most of them came from Fortaleza (55.77%). As for the number of people living at home, there was a median of 3 and an interquartile range (IQR) of 2, a median income of BRL937.00 and an IQR of BRL500.00 (minimum wage during the study period = BRL 954.00/US$ 300.00).

Regarding obstetric data at baseline, most pregnant women were in the second trimester (49.04%), were multiparous (M = 2.8 pregnancies), discovered HIV during the current pregnancy or after the previous pregnancy (61.54%), had no child exposed to HIV (62.5%), and exposed children had a non-reactive diagnosis or were unaware of the diagnosis (69.23%). There was no significant difference in relation to the variables, showing the homogeneity of the groups, including the moment of discovery of HIV.

**Figure 3.**
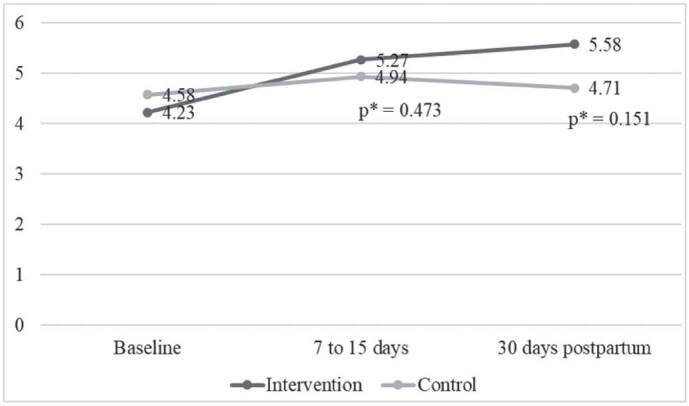
Intergroup comparison of average knowledge level score over Time – Fortaleza, CE, Brazil, 2018.

Knowledge about the prevention of mother-to-child transmission of HIV was measured in the three phases of the study, ranging from zero to 16 points, as shown in Figure 3.

*p = Significance level

Figure 3 shows that there was no difference between the groups, over time, in relation to knowledge, evidencing that the booklet did not bring a significant change in the knowledge of the IG, when compared to the CG.

Complementarily, an intention-to-treat (ITT) analysis was performed, including the 104 pregnant women who started the clinical trial, distributed in both arms of the study, regardless of whether they completed the follow-up period. From the analysis, there were no significant differences regarding the women’s knowledge during the three evaluations, as found in the initial analysis (ITT: baseline p = 0.374; 7–15 days p = 0.837; 30 days postpartum p = 0.468), evidencing that the losses may not have interfered in the result found.

On the other hand, as shown in Table 2, in the intragroup analysis of the average score of knowledge in the three phases of the study, it was found that the intervention with the booklet proved to be efficient in improving the knowledge of pregnant women living with HIV in the IG in the short term (7–15 days; p = 0.002), remaining high in the long term (30 days postpartum; p = 0.033) in relation to the baseline. The CG did not show an increase in the average of knowledge over the time of the study.

**Table 2.** Intra-group comparison of the average score for the level of knowledge over time – Fortaleza, CE, Brazil, 2018. (n = 70)

	Intervention			Control		
	Initial mean (SD*)	Final mean (SD*)	p-value^†^	Initial mean (SD*)	Final mean (SD*)	p-value^†^
**Baseline** **X** **7 to 15 days**	n = 37 4.19 (2.14)	n = 37 5.27 (1.99)	**0.002**	n = 33 4.45 (2.10)	n = 33 4.94 (1.82)	0.051
**Baseline** **X** **30 days**	n = 24 4.71 (2.35)	n = 24 5.58 (1.89)	**0.033**	n = 21 4.10 (2.34)	n = 21 4.71 (2.10)	0.277
**7 to 15 days** **X** **30 days**	n = 24 5.33 (1.69)	n = 24 5.58 (1.89)	0.366	n = 21 4.95 (1.99)	n = 21 4.71 (2.10)	0.620

*SD = Standard Deviation; ^†^ = Student’s t test for paired data.

Similarly, when performing the intention-to-treat analysis in the intragroup comparison, means and p-values were similar to those found in the initial analysis (ITT – INTERVENTION – baseline × 7–15 days p = 0.003; baseline × 30 days p = 0.000; 7–15 days × 30 days p = 0.359/CONTROL – baseline × 7–15 days = 0.051; baseline × 30 days p = 0.376; 7–15 days × 30 days p = 0.613), showing that the losses may not have interfered with the result found.

The study showed an increase in the percentage of mothers in the IG who improved their knowledge about the “use of HIV medication during pregnancy” as a necessary care to prevent vertical transmission, from baseline (71.70%), for the evaluation in the short term of 7–15 days (91.89%; p = 0.004). The variable “use of HIV medication during childbirth” was also well assimilated as a necessary care by the women in the IG, both in the evaluation from baseline (24.53%) to evaluation 2 (51.35%; p = 0.004), and from the baseline for evaluation 3 (66.67%; p = 0.001). There was also an increase in the percentage of women who knew the care to prevent HIV-VT in the postpartum period, from baseline (79.25%) to evaluation 2 (94.59%; p = 0.008). These data reaffirm the booklet’s potential for improving the knowledge of mothers who had access to this technology, especially in the short term.

On the other hand, the care for which the pregnant women showed less knowledge, despite improvement after the intervention, were related to attendance at prenatal consultations, periodic examinations, nutrition and a healthy lifestyle.

## DISCUSSION

The educational booklet proved to be effective, in improving knowledge about the prevention of HIV-VT, in the analysis of the intervention group, especially regarding the use of medication in the gestational, intrapartum and postpartum period. This finding is important, considering that the use of ART is one of the main precautions for the prevention of HIV-VT.

Research carried out in Senegal, with 4,443 children, in which there was an increase in mothers who used adequate treatment (57.4%; N = 2,550) and children who received HIV prophylaxis (52.1%; N = 2315), showed that the transmission rate decreased from 14.8% in 2008 to 4.1% in 2015 (p < 0.001)^(13)^, which reinforces the importance of adherence to pharmacological therapy and consequent effectiveness in reducing VT rates. Therefore, effective adherence to the use of medication starts, initially, from understanding its importance.

In contrast, in this study, women were less aware of care: attendance at prenatal consultations, periodic examinations, healthy eating, and lifestyle. A review research, whose objective was to highlight main care for pregnant women living with HIV, during prenatal care, childbirth, and puerperium, pointed out that, in 23% of the studies, self-care was shown to be beneficial to pregnant women with HIV, because they are sensitized to improve their lifestyle, develop healthy eating habits, besides knowing and controlling the disease-causing risk factors and adopting preventive measures^(14)^.

According to integrative review^(15)^, no publications were found about the construction or use of printed educational materials in the context of HIV-VT, which hinders the comparison of the results of this research with other studies, besides reinforcing the originality and importance of this pilot study.

Thus, it was found that the booklet analyzed had a positive effect on increasing the knowledge of the IG from before to after the educational intervention, in the intragroup analysis, both at 7–15 days and 30 days postpartum. However, in the intergroup analysis, there was no statistical difference between the average knowledge score between the baseline groups for the other evaluations.

Even though the groups were homogeneous at baseline, given the social context of the women involved in this research, the lack of difference between the averages in the intergroup women’s knowledge may be related to factors such as sanitary conditions, housing, hygiene and nutrition, which are also considered determinants for learning^(16)^, not having been deeply evaluated in this pilot study regarding outcomes.

As for the positive effect of the booklet on expanding the knowledge of the intervention group, similarly to the present study, a “before and after” research with educational activity seeking to assess women’s knowledge about prevention, transmission, and perception of vulnerability in relation to Sexually Transmitted Infections (STI) and HIV also found that health education contributed significantly to increasing participants’ knowledge and perception of vulnerability regarding STI/HIV, comparing before-and-after knowledge of the same women^(17)^.

Action research aiming to develop and implement an intervention proposal for health education, through group activities with people living with HIV/AIDS, found that this strategy was a successful experience that could be implemented in health services, with low cost and great potential for impact on building bonds between the health team and users^(18)^. Even though it is a differentiated study, with a greater chance of bonding among the participants, because it is a group study, and without the costs of printed material, as in the case of this pilot, it demonstrates a beneficial effect of health education for the target audience.

One of the health education strategies that have been effective in improving pregnant women’s knowledge are educational booklets. Cluster randomized controlled clinical trial involving 91 pregnant women in the IG and 94 in the CG, proved that the booklet was effective in improving the knowledge, attitude and practice of pregnant women about the use of regional foods (p < 0.001), reinforcing that knowledge is essential for adherence to care^(19)^.

Another successful experience with the use of an educational booklet was demonstrated in a randomized controlled clinical trial with 56 patients in the preoperative period of bariatric surgery, in which the educational intervention mediated by a booklet proved to be more effective in improving the knowledge and maintenance in positive attitude about bariatric surgery, when compared to verbal guidance^(20)^.

Furthermore, the study showed a high sample loss. Surveys that also used the telephone to assess the outcomes of clinical trial-type studies reported significant losses during follow-up, ranging from 35.7% to 56.9%^(12,21)^.

Difficulty in contacting the participants was the main reason for loss of follow-up in the sample. This way, it is clear that, although the telephone is currently an accessible technology and a viable and beneficial strategy for health interventions^(21,22)^, it has limitations, such as the interruption of the follow-up provided due to communication difficulties, as well as the long follow-up time, which included the evaluation of pregnant women until the postpartum period.

It is also inferred that, in addition to the limitation of telephone studies, there is an aggravating factor for this RCT sample loss: involving pregnant and postpartum women with HIV, who are a delicate population, as it carries the stigma of the disease ingrained. Many women do not want to talk about their own condition, they are ashamed. In addition, they may be away from home or close to someone, which makes it impossible to answer the cell phone and makes them more exposed and less available for research.

Added to these situations, losses can also be associated with socioeconomic factors, such as low income, race, and few years of study in the sample. Research that also involved HIV-positive pregnant women points out that socioeconomic factors significantly influence adherence to health care^(23)^. Thus, it is necessary to add a higher percentage of losses for future research involving the monitoring of this public.

Among the limitations of the present study, we can mention the percentage of losses during the follow-up, the peculiar characteristics of this target audience, such as the high stigma related to the infection, which may have contributed to this occurrence, as well as part of the collection having been done through phone calls. In addition, the researchers not being part of the service in which the women performed prenatal care can also be pointed out as a limiting factor that brought resistance from the women to participate and remain in the study. Moreover, some variables could not be controlled, such as the groups receiving extra information throughout the study, the possibility of pregnant women exchanging information among themselves (intergroups), and the fact that the women were not asked, during evaluations 2 and 3, whether they read the booklet after the intervention, which could be associated with knowledge outcomes.

Despite these limitations, regarding the contributions of this RCT pilot study, it is inferred that the study is a pioneering initiative with this audience, who used printed educational material, which allows not only the filling of this gap in the literature, but also the knowledge of the limitations of this study, allowing adjustment of obstacles to the future development of clinical trials, especially in relation to the minimization of losses, such as carrying out in-person sample follow-up and avoiding that part of the collection be done through telephone calls, as well as seeking researchers from the prenatal service who follow the women to have a greater bond and less loss.

## CONCLUSION

This study showed that the booklet “How to prevent the transmission of HIV from mother to child? Be up on it!” increased the levels of short- and long-term knowledge in the intervention group, but there was no difference in the intergroup analysis regarding the knowledge of the participating women. Thus, the booklet can be a strategy to be used with mothers living with HIV, as a way of offering additional information support, so that they can carry out the necessary prevention measures, minimizing the risks of HIV-VT. However, the urgent performance of a broader study based on this pilot one is required, with a larger sample and less loss to follow-up, to prove the effectiveness of the technology used.
